# Equilibrium contrast CMR for the detection of amyloidosis in mice

**DOI:** 10.1186/1532-429X-13-S1-O60

**Published:** 2011-02-02

**Authors:** Adrienne E Campbell, Anthony N Price, Stephan Ellmerich, Paul Simons, Raya Al-Shawi, Philip N Hawkins, Roger J Ordidge, Mark B Pepys, James C Moon, Mark F Lythgoe

**Affiliations:** 1 Centre for Advanced Biomedical Imaging, University College London, London, UK; 2 Robert Steiner MRI Unit, Imperial College London, London, UK; 3 Centre for Amyloidosis and Acute Phase Proteins, Division of Medicine, University College London, London, UK; 4 Department of Medical Physics and Biomedical Engineering, University College London, London, UK; 5 Heart Hospital and Division of Medicine, University College London, London, UK

## Objective

In this study, we optimise equilibrium contrast CMR (EQ-CMR) protocols in mice and apply EQ-CMR to detect AA amyloidosis in the heart and liver of mice with inducible transgenic overexpression of serum amyloid A protein.

## Background

Systematic amyloidosis is a severe, diagnostically challenging, disorder characterised by the extracellular deposition of insoluble abnormal protein fibrils [1]. Recently, Flett et al [2] showed that the volume of distribution of gadolinium (Gd) contrast agents, calculated by EQ-CMR, can be used to measure fibrosis. This technique uses the extracellular nature of Gd to relate the volume of distribution of the agent (V_d_) to extracellular pathology.

## Methods

**Figure 1:**
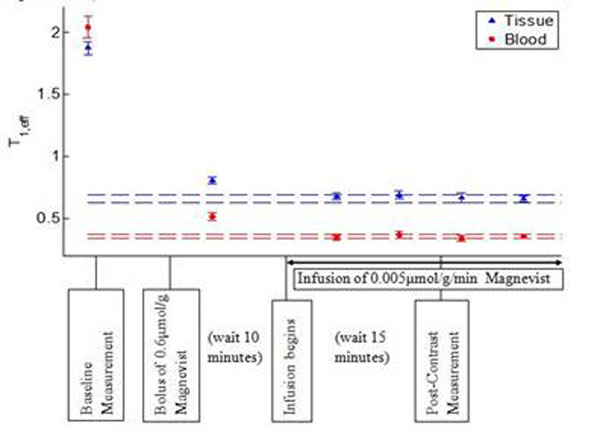
T1,_eff_ plotted to demonstrate the optimized equilibrium protocol in mice. Dotted lines represent values within 5% of final T1 measurement (defined as equilibrium).

A bolus followed by steady infusion of Magnevist was used to generate a blood - tissue equilibrium of [Gd]. The optimal dose and timing protocol, determined empirically, is displayed in Figure 1. An ECG-gated Look-Locker technique [3] was used to measure the T_1_ and the V_d_ can be calculated: V_d_=ΔR_1,tissue_/ ΔR_1,blood_

Nine control and 11 amyloidotic mice [4] (confirmed by histology to have major amyloid deposits in the liver and minor deposits in the heart) were imaged using a standard cine stack and EQ-CMR. A mid-ventricle short-axis slice through the heart, which included a section of liver was used. The hematocrit (Hct) was measured using a blood sample from the tail vein.

## Results

**Figure 2:**
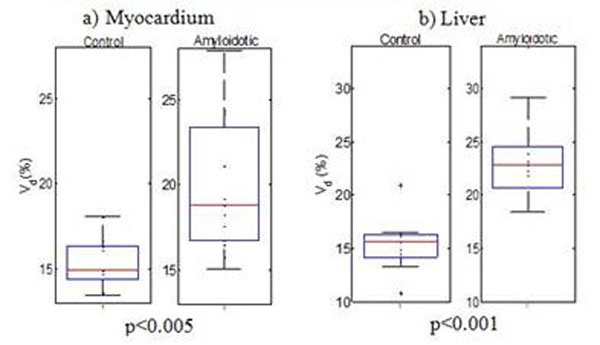
Comparison of V_d_ between groups

Analysis of cardiac functional parameters calculated from cine images showed no significant difference between the groups. Figure 2 presents box-and-whisker plots comparing V_d_ between groups for the (a) myocardium and (b) liver. The amyloidotic group shows a significantly increased V_d_ of Gd compared to the control group in both organs. The V_d_ of the control group was 15.4% ± 0.2% (myocardium) and 15.4±0.3% (liver) and of the amyloidotic group 19.8±0.4% (myocardium) and 23.6± 0.4% (liver) (mean±s.e.m).

## Conclusion

An EQ-CMR procedure has been optimised in the mouse. The results of this study show that EQ-CMR techniques can detect minor amyloid deposits with good sensitivity. This approach has the potential to become a sensitive diagnostic tool with considerable utility in serial quantitative monitoring of response to novel therapy aimed at elimination of amyloid deposits [5,6].
